# Comparison of the Performance of Artificial Intelligence Versus Medical Professionals in the Polish Final Medical Examination

**DOI:** 10.7759/cureus.66011

**Published:** 2024-08-02

**Authors:** Aleksander Jaworski, Dawid Jasiński, Wojciech Jaworski, Aleksandra Hop, Artur Janek, Barbara Sławińska, Lena Konieczniak, Maciej Rzepka, Maximilian Jung, Oliwia Sysło, Victoria Jarząbek, Zuzanna Błecha, Konrad Haraziński, Natalia Jasińska

**Affiliations:** 1 Department of Medicine, Specialist Medical Centre Joint Stock Company, Polanica-Zdrój, POL; 2 Department of Medicine, Prof. K. Gibiński University Clinical Center of the Medical University of Silesia in Katowice, Katowice, POL; 3 Department of Children’s Developmental Defects Surgery and Traumatology, Medical University of Silesia in Katowice, Katowice, POL; 4 Department of Medicine, Fryderyk Chopin University Clinical Hospital in Rzeszów, Rzeszów, POL; 5 Department of Medicine, Medical University of Silesia in Katowice, Katowice, POL; 6 Department of Medicine, Regional Specialised Hospital No. 4 in Bytom, Bytom, POL; 7 Department of Medicine, St. Barbara Specialised Regional Hospital No. 5, Sosnowiec, POL; 8 Department of Medicine, University Clinical Hospital in Opole, Opole, POL; 9 Department of Medicine, Academy of Silesia, Katowice, POL; 10 Department of Cybernetics, Military University of Technology, Warsaw, POL

**Keywords:** medical professionals, medical students, final medical examination, artificial intelligence, machine learning, chatgpt

## Abstract

Background: The rapid development of artificial intelligence (AI) technologies like OpenAI's Generative Pretrained Transformer (GPT), particularly ChatGPT, has shown promising applications in various fields, including medicine. This study evaluates ChatGPT's performance on the Polish Final Medical Examination (LEK), comparing its efficacy to that of human test-takers.

Methods: The study analyzed ChatGPT's ability to answer 196 multiple-choice questions from the spring 2021 LEK. Questions were categorized into "clinical cases" and "other" general medical knowledge, and then divided according to medical fields. Two versions of ChatGPT (3.5 and 4.0) were tested. Statistical analyses, including Pearson's χ^2^ test, and Mann-Whitney U test, were conducted to compare the AI's performance and confidence levels.

Results: ChatGPT 3.5 correctly answered 50.51% of the questions, while ChatGPT 4.0 answered 77.55% correctly, surpassing the 56% passing threshold. Version 3.5 showed significantly higher confidence in correct answers, whereas version 4.0 maintained consistent confidence regardless of answer accuracy. No significant differences in performance were observed across different medical fields.

Conclusions: ChatGPT 4.0 demonstrated the ability to pass the LEK, indicating substantial potential for AI in medical education and assessment. Future improvements in AI models, such as the anticipated ChatGPT 5.0, may enhance further performance, potentially equaling or surpassing human test-takers.

## Introduction

In recent years, artificial intelligence (AI), machine learning, deep learning, and natural language processing have been increasingly employed across various fields of medicine. The use of AI in healthcare is rapidly expanding in areas such as predicting mortality in intensive care patients, drug development, and forecasting the success of surgical procedures [1]. The number of companies involved in the process of its development grows constantly. One among them is OpenAI.

OpenAI designed the Generative Pretrained Transformer (GPT), and its variant ChatGPT is a leading AI model based on machine learning. It uses deep learning techniques and natural language output data to generate human-like responses. Since its public release in November 2022, the program gained one million users in just five days, and currently, it has 180 million registered users, with 12% of them being Americans [2]. The number of visits to the openai.com website in February 2024 alone exceeded 1.6 billion entries. It is estimated that 100 million users use ChatGPT every week [3].

To achieve similar results, tech giants, social media platforms, and service companies like Instagram, Facebook, Twitter (X), Airbnb, and Netflix had to work for approximately 2.5, 10, 24, 30, and 42 months, respectively. The only application that surpassed this achievement was Threads, which gathered one million users in just one hour [4].

ChatGPT was trained on a massive amount of text data, exceeding 45 terabytes [5]. The ChatGPT language model uses deep neural networks to analyze and generate texts based on the data inputted into the model. Currently, the United States is increasingly implementing ChatGPT's functionality in medicine. A study was even conducted there to demonstrate ChatGPT's effectiveness in solving exam tasks from the United States Medical Licensing Exam (USMLE). The study results were surprising, as the model achieved a score of 60%, which resulted in passing the exam [6].

In Poland, this tool has not yet been trained to describe patients more effectively, provide medical education, and gather medical information [7]. Kufel et al. tested ChatGPT's effectiveness in the context of writing the national radiology specialization exam. Although the model failed, in some subcategories, it came close to achieving the passing threshold [8]. National specialization exams, depending on the medical field, vary in difficulty and question detail, but regardless of the field, they are presumably more challenging than the Final Medical Examination (Lekarski Egzamin Końcowy, LEK).

Our study aimed to assess ChatGPT's effectiveness in answering questions from the LEK and analyze its strengths and weaknesses compared to human cognition.

## Materials and methods

The study was conducted between April 12 and April 30, 2024. It involved analyzing the spring 2021 edition of LEK, which was randomly selected from previous exams in the question database of the Center for Medical Education (Centrum Egzaminów Medycznych, CEM) in Łódź, Poland. The selected exam initially consisted of 200 multiple-choice questions, each with one correct answer among five distractors, chosen using a random number generator. Three questions were withdrawn by CEM due to lacking a clear correct answer or being inconsistent with current medical knowledge. One question involved interpreting an ECG reading this question was excluded due to ChatGPT 3.5's inability to evaluate graphics.

To ensure a comprehensive analysis of all questions, they were divided into those related to "clinical cases," where the correct answer had to be chosen on the basis of the clinical description of a specific patient, and "other" questions related to general medical knowledge. Additionally, all questions were categorized according to medical fields in line with the LEK division, i.e., questions from internal medicine, pediatrics, surgery, gynecology and obstetrics, psychiatry, emergency medicine and intensive care, family medicine, bioethics and medical law, medical certification, and public health. Two independent researchers conducted the categorization, which was later accepted by a third independent researcher. Due to the multicenter characteristics of the study, authors cooperated via remote communication methods, such as Microsoft Teams, Zoom, Facebook Messenger, emails, and Google Docs. All sections of the study prepared by the individual groups were reviewed by the other authors, allowing each researcher to contribute to every part of the study using the aforementioned remote communication methods.

Data collection and analysis

Before introducing ChatGPT to the questions, it was familiarized with the exam regulations, which included information about the number of questions, the number of possible answers, and the number of correct answers. Furthermore, after each question was input into the model, ChatGPT was asked, "On a scale of one to five, how confident did you feel about the question?" This question aimed to gauge ChatGPT's confidence level in selecting answers. ChatGPT could respond to this confidence question as follows: one - unsure, two - not very sure, three - almost sure, four - very sure, five - completely sure. All questions were input into ChatGPT, and all interactions with it were documented. To maintain consistency with the content of the LEK exam questions, the entire interaction with ChatGPT was conducted in Polish. The study used versions 3.5 and 4.0 of ChatGPT.

Statistical analysis

The results obtained from ChatGPT were compared with the correct answers recognized by CEM in Łódź. The assessment of ChatGPT's effectiveness involved determining the percentage of correct answers provided by ChatGPT (divided according to medical fields). ChatGPT's confidence in giving both correct and incorrect answers was also analyzed.

Pearson's chi-square test was used to assess the relationship (significance) between the distribution of correct and incorrect answers, question type, and other qualitative variables. The Mann-Whitney U test was used to compare qualitative variables between groups. The STATISTICA program was used for statistical analysis. Additionally, to compare the confidence level between correct and incorrect answers, the Mann-Whitney U test was applied. P-values less than 0.05 were considered statistically significant.

## Results

ChatGPT 3.5 answered 99 questions correctly (50.51%) and 97 questions incorrectly (49.49%). ChatGPT 4.0 answered 152 questions correctly (77.55%) and 44 questions incorrectly (22.45%) (Table 1). Considering the division of questions into "other" and "clinical cases," version 3.5 answered 88 "other" questions correctly (52.07%) and 81 incorrectly (47.93%), and 11 clinical case questions correctly (40.74%) and 16 incorrectly (59.26%). Version 4.0 answered 133 "other" questions correctly (78.7%), 36 incorrectly (21.3%), and 19 clinical case questions correctly (70.37%) and 8 incorrectly (29.63%) (Table 2).

**Table 1:** General summary of ChatGPT 3.5 and ChatGPT 4.0 results

ChatGPT version	Correct answers n (%)	Incorrect answers n (%)
ChatGPT 3.5	99 (50.51)	97 (49.49)
ChatGPT 4.0	152 (77.55)	44 (22.45)

**Table 2:** ChatGPT 3.5 and ChatGPT 4.0 results in “clinical cases” and “other” questions

ChatGPT version	Correct answers n (%)	Incorrect answers n (%)	p-value
ChatGPT 3.5			
Clinical cases	11 (40.74)	16 (59.26)	p = 0.376
Other	88 (52.07)	81 (47.93)	
ChatGPT 4.0			
Clinical cases	19 (70.37)	8 (29.63)	p = 0.475
Other	133 (78.70)	36 (21.30)	

From various fields of medicine, the ChatGPT 3.5 answers were: bioethics and medical law 6 correct answers (60%), and 4 incorrect answers (40%); surgery 14 correct answers (51.85%), and incorrect answers 13 (48.15%); internal medicine 21 correct answers (53.85%), and 18 incorrect answers (46.15%); family medicine 12 correct answers (63.16%), 7 incorrect answers (36.84%); emergency medicine 8 correct answers (44.44%), and incorrect answers 10 (55.56%); medical certification 2 correct answers (28.57%), and 5 incorrect answers (71.43%); Pediatrics 10 correct answers (34.48%), and 19 incorrect answers (65.52%); obstetrics and gynecology 9 correct answers (34.62%), and 17 incorrect answers (65.38%); psychiatry 11 correct answers (78.57%), 3 incorrect answers (21.43%); and public health 6 correct answers (85.71%), and 1 incorrect answer (14.29%).

Analogously for the ChatGPT 4.0 version

From various fields of medicine, the ChatGPT 4.0 answers were: bioethics and medical law 7 correct answers (70%), and 3 incorrect answers (30%); surgery 21 correct answers (77.78%), and incorrect answers 6 (22.22%); internal medicine 33 correct answers (84.62%), and 6 incorrect answers (15.38%); family medicine 15 correct answers (78.95%), 4 incorrect answers (21.05%); emergency medicine 16 correct answers (89.47%), and incorrect answers 2 (10.53%); medical certification 4 correct answers (57.14%), and 3 incorrect answers (42.86%); pediatrics 19 correct answers (65.52%), and 10 incorrect answers (34.48%); obstetrics and gynecology 21 correct answers (80.77%), and 5 incorrect answers (19.23%); psychiatry 9 correct answers (64.29%), 5 incorrect answers (35.71%); public health 7 correct answers (100%), and 0 incorrect answers (0%).

The overall percentage of correct and incorrect answers to questions from various fields of medicine, including the percentage values for ChatGPT 3.5, is presented in Table 3 and for ChatGPT 4.0 in Table 4. The chi-squared test results for the distribution of questions by specific medical fields showed p-values of approximately 0.24 for ChatGPT 3.5 and 0.26 for ChatGPT 4.0. For the division into clinical cases and other questions, the p-value was 0.376 for ChatGPT 3.5 and 0.475 for ChatGPT 4.0, suggesting no significant differences between the groups.

**Table 3:** ChatGPT 3.5 results in specific medical fields

Medical field	Correct answers n (%)	Incorrect answers n (%)	p-value
Bioethics and medical law	6 (60)	4 (40)	p = 0.24
Surgery	14 (51.85)	13 (48.15)	
Internal medicine	21 (53.85)	18 (46.15)	
Family medicine	12 (63.16)	7 (36.84)	
Emergency medicine	8 (44.44)	10 (55.56)	
Medical certification	2 (28.57)	5 (71.43)	
Pediatrics	10 (34.48)	19 (65.52)	
Obstetrics and gynecology	9 (34.62)	17 (65.38)	
Psychiatry	11 (78.57)	3 (21.43)	
Public health	6 (85.71)	1 (14.29)	

**Table 4:** ChatGPT 4.0 results in specific medical fields

Medical field	Correct answers n (%)	Incorrect answers n (%)	p-value
Bioethics and medical law	7 (70)	3 (30)	p = 0.26
Surgery	21 (77.78)	6 (22.22)	
Internal medicine	33 (84.62)	6 (15.38)	
Family medicine	15 (78.95)	4 (21.05)	
Emergency medicine	16 (89.47)	2 (10.53)	
Medical certification	4 (57.14)	3 (42.86)	
Pediatrics	19 (65.52)	10 (34.48)	
Obstetrics and gynecology	21 (80.77)	5 (19.23)	
Psychiatry	9 (64.29)	5 (35.71)	
Public health	7 (100)	0 (0)	

**Figure 1:**
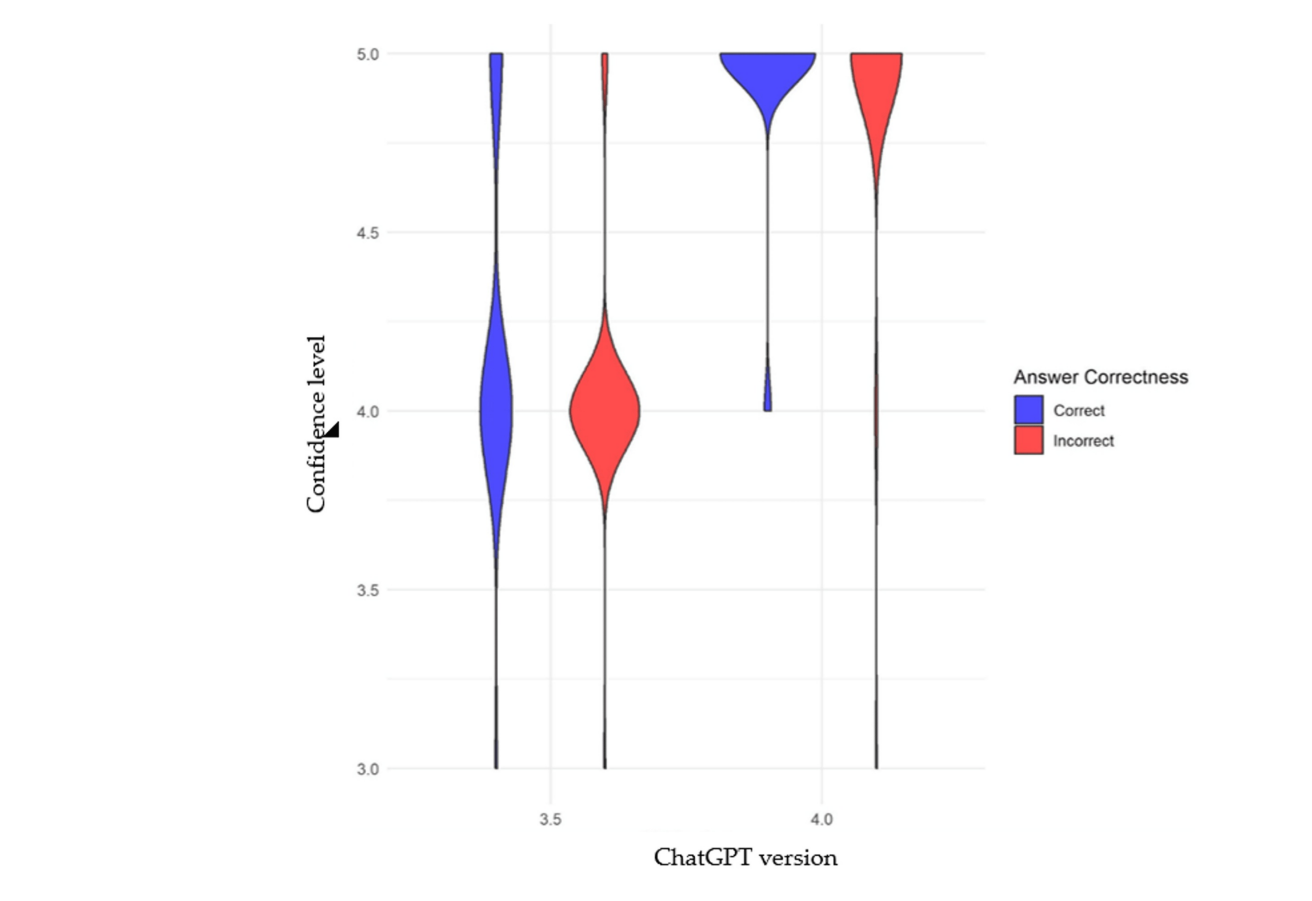
Comparison of confidence levels for correct and incorrect answers for both ChatGPT versions ChatGPT 3.5 The p-value is 0.0034. We can conclude that confidence levels for correct answers are significantly different from confidence levels for incorrect answers. Such a difference may suggest that AI is more confident in its correct answers compared to its incorrect answers, or vice versa. ChatGPT 4.0 The p-value is 0.93. We can conclude that the confidence levels for correct answers are not significantly different from the confidence levels for incorrect answers. Such a result may suggest that AI has similar confidence levels regardless of whether the answer is correct or incorrect.

In the Mann-Whitney test analyzing the confidence in both correct and incorrect answers, version 3.5 had a p-value of 0.0034, and version 4.0 had a p-value of 0.93, indicating a significant difference in confidence for version 3.5 but not for version 4.0 (Figure 1).

## Discussion

AI is a field of computer science dedicated to creating machines, systems, and computer programs with computational capabilities that enable them to think, learn, and make decisions in a human-like manner [9]. AI has found applications in areas such as natural language processing, image recognition, industry, finance and banking, customer service, education, and medicine [10]. Its development in medicine has the potential to transform the way healthcare professionals work and communicate with patients. AI in medicine is primarily used in diagnostics, personalized healthcare, screening and prevention, administrative process automation, research coordination, and medical education [11].

These applications of AI in medicine are just examples, and AI has enormous potential to continually increase its utility in the future as it evolves. However, using AI also comes with certain risks that need to be managed. The main risks associated with using AI in medicine include data privacy and security, the increased frequency of cyberattacks, accountability for decisions made by AI, displacement of healthcare workers, and dependency on technology [12].

LEK is a test consisting of 200 multiple-choice questions with five answer options each. The test has a fixed distribution of questions from various medical fields, including 39 questions from internal medicine, 29 from pediatrics, 27 from surgery, 26 from obstetrics and gynecology, 14 from psychiatry, 20 from family medicine, 20 from emergency medicine and intensive care, 10 from bioethics and medical law, seven from medical certification, and 8 from public health. In total, 20 questions must relate to oncology. Test-takers have four hours to complete the exam. Over the past few years, the format of the exam has undergone significant changes, the most notable being the introduction of a publicly available question bank, with 70% of the exam questions coming from this bank starting from the autumn 2021 edition. This change has significantly improved the pass rate of the exam, which previously saw 20-30% of candidates failing, now down to 1-3% [13,14].

The introduction of the question bank also leveled the playing field among test-takers. The LEK score is crucial for medical specialization recruitment in Poland, making this change in exam format a major disadvantage for many doctors in the recruitment process. Besides mastering the publicly available question bank, having adequate knowledge, and a significant element of luck now play a larger role in passing the exam. To pass the exam, a score of 56% is required. This means that any candidate who has thoroughly memorized the question bank should score at least 14% above the passing threshold. Such a low pass threshold questions the validity of LEK as a criterion for obtaining full professional practice rights and as a key element in the recruitment process for medical specializations.

Our study aimed to determine whether a machine learning-based AI algorithm could pass the exam without prior memorization of exam questions for two reasons: first, to assess AI's capabilities in solving complex, nuanced medical questions; and second, to address recent reports suggesting that exam organizers are considering moving away from the publicly available question bank in the near future.

ChatGPT version 3.5 did not achieve the required 56% of correct answers, falling short by about 5.5%. Notably, this version of ChatGPT was significantly more confident in its correct answers than its incorrect ones. ChatGPT version 4.0, however, scored over 77.5% on the LEK, meaning the newer version passed the exam with a 20% margin. Additionally, its confidence in answering questions did not significantly differ between correct and incorrect answers.

In the spring 2021 session of the LEK, 4779 people took the exam. The highest score was 196 out of 200 points, and the lowest was 54. The exam was passed by 4700 individuals, with 79 failing, resulting in a failure rate of over 1.69%. The score achieved by ChatGPT 4.0 was significantly below the average scores obtained by students or graduates of various medical schools. Considering that test-takers knew 70% of the questions in advance, and ChatGPT did not, the authors of this study find ChatGPT's performance to be at least impressive. Before the era of the publicly available question bank, the average scores of graduates from various medical schools ranged from 60% to 70%. The score achieved by ChatGPT 4.0 would have been considered relatively high at that time.

ChatGPT version 4.0 debuted on March 13, 2023, representing a significant leap over version 3.5, which debuted in November 2022. The main difference between ChatGPT-4 and ChatGPT-3.5 is ChatGPT-4's ability to analyze and recognize images and graphics [15]. This capability led us to manually exclude the EKG analysis question to standardize the comparison between the two AI versions. Model 3.5 focused mostly on text processing, while 4.0 can understand images, enabling better interactions with users. Version 4.0 also has a larger memory than its predecessor, remembering up to 50 pages of text or up to 64,000 words, allowing the chatbot to refer to past events. ChatGPT 4.0 also significantly expanded its linguistic capabilities, providing answers to thousands of multiple-choice questions in 26 different languages. Additionally, it has been optimized for spelling and grammar accuracy, which we believe contributed to better interpretation of LEK questions [14]. Given that there was only a six-month gap between the releases of both versions, it can be stated that the technology has made incredible progress. Version 5.0 is expected to debut in 2024 and is anticipated to be at least as improved over version 4.0 as version 4.0 was over version 3.5.

The differences observed between versions 3.5 and 4.0 in our study prompt us to consider the potential of the upcoming version 5.0. It raises the question of whether version 5.0 will meet or even surpass the highest scores achieved by medical students and graduates.

It is noteworthy to mention the limitations of this study which are a relatively small number of questions from each medical field that make statistical analysis less reliable and the lack of the ability to asses ECG diagrams by ChatGPT 3.5 which forced us to manually withdraw one question from the data.

## Conclusions

ChatGPT is a powerful tool that has yet to reach its full potential. The currently available versions, 3.5 and 4.0, have demonstrated high effectiveness and confidence in answering test questions from the LEK exam. Version 4.0 proved the capability of passing the LEK with a score similar to the average student or junior medical doctor without access to a public question bank.

The lack of a statistically significant difference in the level of difficulty of questions from different medical fields for both ChatGPT versions can be the result of a limited total number of questions included. It would be crucial to conduct another study with a larger sample of questions to determine whether this difference truly exists.
